# Metagenomic next-generation sequencing, instead of procalcitonin, could guide antibiotic usage in patients with febrile acute necrotizing pancreatitis: a multicenter, prospective cohort study

**DOI:** 10.1097/JS9.0000000000001162

**Published:** 2024-02-09

**Authors:** Chiayen Lin, Jiarong Li, Baiqi Liu, Xiaoyue Hong, Tao Luo, Jinsong Ye, Yi Yu, Xinran Peng, Shanmiao Gou, Huayong Tang, Tongli Yuan, Jianguan Luo, Ming Yang, Bin Feng, Zhijian Zhao, Caihong Ning, Zefang Sun, Shuai Zhu, Lu Chen, Dingcheng Shen, Gengwen Huang

**Affiliations:** a Department of Pancreatic Surgery, General Surgery; b Department of Hernia and Abdominal Wall Surgery; c National Clinical Research Center for Geriatric Disorders, Xiangya Hospital, Changsha; d Department of General Surgery, Changde Hospital, Xiangya School of Medicine, Central South University, Changde; e Department of Hepatobiliary Surgery, General Surgery, The First People’s Hospital of Chenzhou City, Chenzhou; f Department of Pancreatic Surgery, General Surgery, Union Hospital, Tongji Medical College, Huazhong University of Science and Technology, Wuhan; g Department of General Surgery, The First Affiliated Hospital of Hunan Traditional Chinese Medical College; h Department of Hepatobiliary Surgery, General Surgery, Liuyang People’s Hospital, Changsha; i Center of Hepatobiliary and Pancreatic Surgery, Zhuzhou Hospital Affiliated to Xiangya School of Medicine, Central South University, Zhuzhou, Hunan Province, People’s Republic of China

**Keywords:** acute necrotizing pancreatitis, antibiotic stewardship, infected pancreatic necrosis, metagenomic next-generation sequencing, procalcitonin

## Abstract

**Backgrounds:**

The effectiveness of procalcitonin-based algorithms in guiding antibiotic usage for febrile acute necrotizing pancreatitis (ANP) remains controversial. Metagenomic next-generation sequencing (mNGS) has been applied to diagnose infectious diseases. The authors aimed to evaluate the effectiveness of blood mNGS in guiding antibiotic stewardship for febrile ANP.

**Materials and methods:**

The prospective multicenter clinical trial was conducted at seven hospitals in China. Blood samples were collected during fever (T ≥38.5°C) from ANP patients. The effectiveness of blood mNGS, procalcitonin, and blood culture in diagnosing pancreatic infection was evaluated and compared. Additionally, the real-world utilization of antibiotics and the potential mNGS-guided antimicrobial strategy in febrile ANP were also analyzed.

**Results:**

From May 2023 to October 2023, a total of 78 patients with febrile ANP were enrolled and 30 patients (38.5%) were confirmed infected pancreatic necrosis (IPN). Compared with procalcitonin and blood culture, mNGS showed a significantly higher sensitivity rate (86.7% vs. 56.7% vs. 26.7%, *P*<0.001). Moreover, mNGS outperformed procalcitonin (89.5 vs. 61.4%, *P*<0.01) and blood culture (89.5 vs. 69.0%, *P*<0.01) in terms of negative predictive value. Blood mNGS exhibited the highest accuracy (85.7%) in diagnosing IPN and sterile pancreatic necrosis, significantly superior to both procalcitonin (65.7%) and blood culture (61.4%). In the multivariate analysis, positive blood mNGS (OR=60.2, *P*<0.001) and lower fibrinogen level (OR=2.0, *P*<0.05) were identified as independent predictors associated with IPN, whereas procalcitonin was not associated with IPN, but with increased mortality (Odds ratio=11.7, *P*=0.006). Overall, the rate of correct use of antibiotics in the cohort was only 18.6% (13/70) and would be improved to 81.4% (57/70) if adjusted according to the mNGS results.

**Conclusion:**

Blood mNGS represents important progress in the early diagnosis of IPN, with particular importance in guiding antibiotic usage for patients with febrile ANP.

## Introduction

Highlights

- Blood metagenomic next-generation sequencing showed superior diagnostic performance for infected pancreatic necrosis compared with procalcitonin.
- Real-world antibiotic utilization in febrile acute necrotizing pancreatitis was not ideal.
- The metagenomic next-generation sequencing-guided antimicrobial therapy could potentially improve the rate of correct antibiotic usage to 81.4% for patients with febrile acute necrotizing pancreatitis.

Acute pancreatitis (AP) is one of the leading causes of emergency admissions among gastrointestinal disorders, resulting in significant utilization of healthcare resources^1^. The disease is characterized with local and systemic inflammatory response and has a varying clinical course. Approximately 20% of patients develop acute necrotizing pancreatitis (ANP), which involves the necrosis of pancreatic or peripancreatic tissue. Infected pancreatic necrosis (IPN) occurs in 20–40% of ANP and is associated with a high mortality (30–40%)^2^. Early diagnosis of IPN remains a great challenge to the clinicians since sterile ANP and IPN may share similar clinical manifestations. Therefore, the indiscriminate use of antibiotics in ANP is quite common, which inevitably results in the emergence of antimicrobial resistance, unnecessary drug adverse events, and higher healthcare expenses^3–5^. Moreover, it has been established that infection caused by multidrug-resistant organisms is associated with adverse outcomes and increased mortality among patients with IPN^6^.

Thus, it is imperative to identify a simple and effective method for diagnosing pancreatic infection and guiding appropriate antibiotic usage. Fine needle aspiration (FNA) has been previously used as the procedure of choice to establish the diagnosis of pancreatic infection. Unfortunately, the demand for high standard technique and personal experience as well as the potential risk of iatrogenic complications do not render FNA an ideal approach^7,8^. Although air bubble sign within the necrotic collection presented on computed tomography is considered proof of infection, it occurs only in a minority of cases (20% or less) with infected necrosis^9^. In recent years, great expectations have been placed on procalcitonin which acts as a sensitive biomarker of bacterial infections^10–12^. The results of a single-center, patient-blinded, randomized controlled trial (PROCAP) indicate that procalcitonin-guided care may reduce the use of antibiotics without increasing the risk of infection in patients with AP^13^. However, there is no statistically significant risk difference of antibiotic use in patients with moderate or severe AP who had significantly higher risk of being infected^14,15^. Thus, there is an urgent need for an effective and easily accessible infection guide that can accurately identify patients who develop pancreatic infections. Such a guide would significantly improve the outcomes of ANP.

Metagenomic next-generation sequencing (mNGS) is a culture-independent analysis that uses sequencing technologies to study a mixture of microbial genomes. Numerous published case reports and clinical studies have demonstrated the successful application of mNGS in various sample types, including blood, urine, feces, cerebrospinal fluid, respiratory secretions, and tissues. In theory, this high-throughput technique has the potential to identify all pathogens present in a clinical sample through a single sequencing run, providing valuable evidence for guiding treatment options and improving antibiotic stewardship. Significantly, mNGS offers notable advantages in diagnosing infections that are rare or difficult to detect, as well as those involving novel or complex pathogens. Additionally, it has shown efficacy in diverse infectious disorders, such as pancreatic infections, and is rapidly transitioning from research settings to clinical laboratories^16–18^. Our prospective single-center study has shown the potential value of blood mNGS in diagnosing IPN with affordable cost and shorter turnaround time^18^. mNGS of pancreatic fluid aspiration has also been investigated in a small cohort of suspected IPN^19^. However, there is no prospective multicenter study to provide a more thorough insight into the implications of mNGS in the diagnosis of IPN and especially the guidance of antibiotics usage.

We designed this prospective multicenter trial specifically targeting patients with febrile ANP who were usually administered antibiotics indiscriminately in clinical practice. In this study, the effectiveness of blood mNGS, procalcitonin, and blood culture in diagnosing pancreatic infection was evaluated and compared. Additionally, the real-world utilization of antibiotics was also analyzed. Our hypothesis was that mNGS, instead of procalcitonin, could guide antibiotics usage in patients with febrile ANP.

## Methods

### Study design

This study was a prospective, multicenter, observational clinical trial that was conducted at seven hospitals in China from May 2023 to October 2023. The cohort included clinical, radiological, microbiological, and follow-up data. The study protocol was reviewed and approved by the ethics committee of all the participating hospitals. Written informed consent was obtained from all patients or their representative for the study participation. This study has been reported in line with the STROCSS (Strengthening the Reporting of cohort, cross-sectional and case-control studies in Surgery) Guideline^20^ (Supplemental Digital Content 1, http://links.lww.com/JS9/B880). The study was performed in accordance with the Declaration of Helsinki.

### Definition and patient selection

The diagnosis and classification of AP and ANP were based on the Revised Atlanta Classification^21^ and the American Gastroenterological Association guideline^7^. Persistent organ failure (POF) was defined as that at least one of the three organ systems (pulmonary, circulatory, and renal) failure lasted for 48 h or more. IPN was defined as the presence of positive culture in pancreatic or peripancreatic necrotic specimens obtained during the initial drainage procedure. Sterile pancreatic necrosis (SPN) was defined as negative culture in pancreatic or peripancreatic specimens during the initial surgical intervention, or as the absence of infection signs upon discharge or follow-up without the necessity of any further surgical intervention. Antibiotic susceptibility was defined as reported by the local microbiology laboratories. When a susceptibility report was missing, the susceptibility was additionally interpreted by a clinical microbiologist according to the European Committee on Antimicrobial Susceptibility Testing (EUCAST) guidelines^22^.

Patients with febrile ANP, which were defined as clinical manifestation of sepsis with temperature ≥38.5°C at least once during disease course without prior abdominal or retroperitoneal invasive procedure, were enrolled in this study. The exclusion criteria included age under 14 years old and confirmed extra-pancreatic infectious complications at screening, such as biliary infection, urinary tract infection, and pneumonia.

### Sample collection and data acquisition

Blood samples were collected simultaneously for mNGS, procalcitonin, culture, and other routine biomarkers such as C-reaction protein during fever (T ≥38.5°C). For patients who met surgical indications, peripancreatic specimens were collected for microbial culture during the initial surgical intervention.

The mNGS testing and bioinformatic analysis were provided by BGI China. The mNGS protocol has been described previously^18^. After quality control, ≥1 reads of bacteria or fungi detected by mNGS were considered positive. Procalcitonin assay and microbial cultures were performed according to the standard protocols in each study center. Procalcitonin was analyzed by a semi-automated chemoluminescent immunoassay (LUMITEST-PCT, BRAHMS Diagnostica AG). In accordance with Siriwardena’s study^13^, the threshold for positive procalcitonin in this study was 1.0 ng/ml. The fully automated blood culture apparatus was BACTECFX (Becton, Dickinson and Company). Bacterial identification was performed by using the VITEK MS detection system (BioMérieux, Marcyl’Étoile). A positive culture was defined as the detection of a specific pathogen such as bacteria or fungi in the specimen, while a negative culture was defined as the absence of any organisms growing during the incubation period.

The data of clinical manifestations, laboratory tests, imaging results, antibiotic usage, and clinical outcomes were extracted from electronic medical records.

### Treatment modality

This study was a real-world observational trial. The results of mNGS were blinded to the multidiscipline teams consisting of pancreatic surgeons, intensive care physicians, gastroenterologists, and interventional radiologists. Broad-spectrum antibiotics with the ability to penetrate pancreas were administered to patients with febrile ANP when the body temperature was greater than 38.5°C. If the antibiotics treatment failed, such as persistent fever without resolution or development of POF, a step-up surgical approach consisting of percutaneous catheter drainage, minimal access retroperitoneal necrosectomy, and/or open necrosectomy would be attempted.

### Statistical analysis

Continuous variables were presented as mean± SD and median (interquartile range, IQR) depending on the distribution. Categorical variables were described as *n* (%). *χ* ^2^ test or Fisher exact test was used to compare the distribution of categorical variables, and Student *t*-test or Mann–Whitney *U* test for continuous variables. The primary endpoint was the diagnosis of either IPN or SPN. The secondary endpoint was the mortality. Sensitivity, specificity, positive predictive value (PPV), negative predictive value (NPV), and diagnostic accuracy (the sum of true positives and true negatives divided by total) were calculated and used to examine the performance of blood mNGS, procalcitonin, and blood culture for diagnosing IPN using the Pearson *χ* ^2^ test. In the univariate analysis, Student *t*-test, Fisher exact test, Mann–Whitney *U* test, and *χ* ^2^ test were used for bivariate comparisons. The significant variables were subsequently included in the multivariate analysis to identify independent risk factors. This was achieved using binary logistic regression analysis. Odds ratio (OR) with 95% CI was calculated. Data analyses were performed using SPSS statistics, version 22 (IBM Corp) and GraphPad Prism 10 (GraphPad Software). A two-sided *P* value <0.05 was considered statistically significant.

## Results

### Clinical characteristics and outcomes

**Figure 1:**
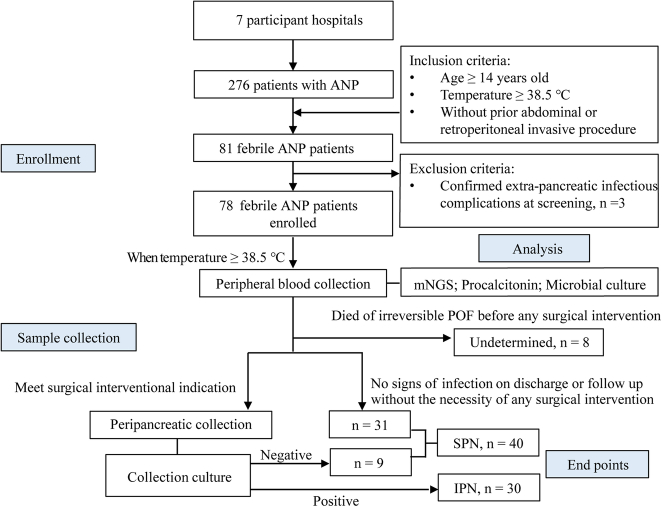
Study flow diagram. ANP, acute necrotizing pancreatitis; IPN, infected pancreatic necrosis; mNGS, metagenomic next-generation sequencing; POF, persistent organ failure; SPN, sterile pancreatic necrosis.

Between May 2023 and October 2023, a total of 81 patients with febrile ANP were prospectively and consecutively enrolled from a pool of 276 ANP patients. Three patients were excluded from the analysis due to confirmed biliary infection (*n*=1), urinary infection (*n*=1), and pulmonary infection (*n*=1), resulting in a final sample size of 78 patients. The flow chart for patient selection was described in Figure 1. Baseline characteristics, major complications, interventions, and clinical outcomes of the patients were summarized in Table 1. The median age was 45.0 years (33.5–51.5 years) with 58 (74.4%) males. The median time from onset to the manifestation of fever (also the blood sample collection) was 10.5 days (6.0–17.8 days). IPN was confirmed in 30 out of 78 patients (38.5%), with a median onset time of 15 days (9.3–19.8 days). The peripancreatic cultures in the patients with IPN revealed polymicrobial infections in ten patients (33.3%). Among the 30 patients with IPN, gram-negative bacteria were isolated in 22 patients (73.3%) by culture, with *Acinetobacter baumannii* being the most frequently reported pathogen (*n*=9, 30.0%). Gram-positive bacteria were isolated in 12 patients (40.0%), with *Enterococcus faecium* being the most frequent species (*n*=10, 33.3%). Fungal infections were found in five patients (16.7%), with *Candida albicans* being the most frequently reported fungus species (*n*=2, 6.7%). Eight patients (10.3%) who died of irreversible POF before any surgical intervention could be attempted were classified undetermined, since FNA was never used to confirm IPN in participating hospitals. The remaining 40 patients (51.3%) were classified as SPN, among which 31 patients (77.5%) did not undergo any surgical intervention. During hospitalization, the overall mortality rate of the entire cohort was 20.5% (16/78).

**Table 1:** Clinical characteristics and outcomes in 78 patients with febrile ANP.

	Overall (*n*=78)
Age, years	45.0 (33.5–51.5)
Male sex	58 (74.4)
Etiology	
Hyperlipidemia	45 (57.6)
Biliary	25 (32.1)
Alcoholic	2 (2.6)
Other	6 (7.7)
Severity	
Moderate severe pancreatitis	43 (55.1)
Severe pancreatitis	35 (44.9)
Laboratory values	
Leukocytes, 10^9^/l	12.8 (8.3–17.0)
CRP, mg/l	149.0 (106.5–203.8)
Procalcitonin, ng/ml	0.8 (0.3–6.4)
Fibrinogen, g/l	5.2 (4.1–6.7)
Blood urea nitrogen, mmol/l	6.2 (3.8–11.4)
Air bubble sign	6 (7.7)
Positive blood mNGS	38 (48.7)
Positive procalcitonin	35 (44.9)
Positive blood culture	16 (20.5)
ICU-admission	36 (46.2)
Length of ICU stay, days	7.5 (4.0–14.5)
Length of hospital stay, days	17.0 (11.5–27.0)
Empirical antibiotics usage	72 (92.3)
Surgical interventions	40 (51.3)
Step-up approach	32 (80.0)
Complications	
Intestinal fistula	1 (1.3)
Intra-abdominal hemorrhage	2 (2.6)
Pancreatic fistula	4 (5.1)
Deep vein thrombosis	11 (14.1)
IPN	30 (38.5)
Death	16 (20.5)

Data are presented as *n* (%) or median (IQR).

ANP, acute necrotizing pancreatitis; CRP, c-reactive protein; IPN, infected pancreatic necrosis; mNGS, metagenomic next-generation sequencing.

### Diagnostic performance of blood mNGS, procalcitonin, and blood culture for differentiating IPN from SPN

**Figure 2:**
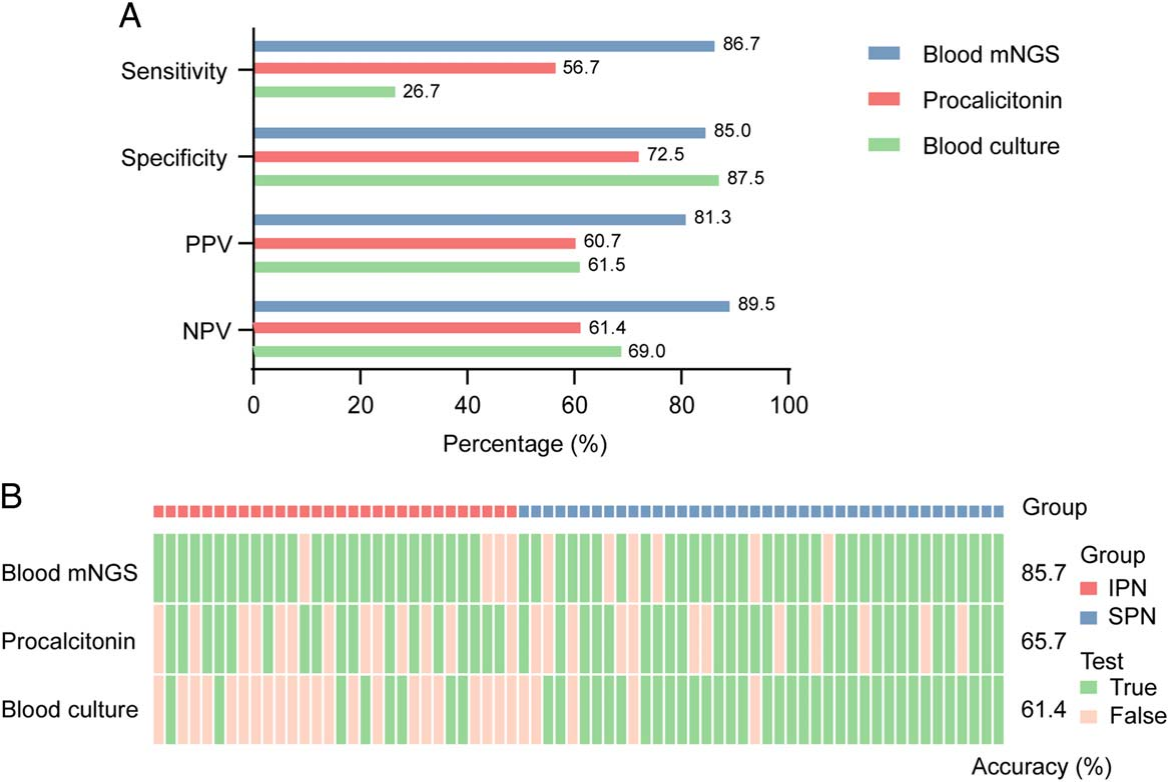
A. Diagnostic performance of blood mNGS, procalcitonin, and blood culture in diagnosing IPN; B. Diagnostic accuracy of blood mNGS, procalcitonin, and blood culture in diagnosing IPN and SPN. IPN, infected pancreatic necrosis; mNGS, metagenomic next-generation sequencing; NPV, negative predictive value; PPV, positive predictive value; SPN, sterile pancreatic necrosis.

The sensitivity, specificity, PPV, and NPV of blood mNGS, procalcitonin, and blood culture for diagnosing IPN were illustrated in Figure 2A. Compared with procalcitonin and blood culture, mNGS showed a significantly higher sensitivity rate (86.7% vs. 56.7% vs. 26.7%, *P*<0.001). Moreover, mNGS outperformed procalcitonin (89.5 vs. 61.4%, *P*<0.01) and blood culture (89.5 vs. 69.0%, *P*<0.01) in terms of NPV. However, there were no statistically significant differences in terms of specificity and PPV among the three methods. The overall diagnostic accuracy of blood mNGS, procalcitonin, and blood culture was presented in Figure 2B. Notably, blood mNGS exhibited the highest accuracy (85.7%) in diagnosing IPN and SPN, significantly superior to both procalcitonin (65.7%) and blood culture (61.4%).

### Predictors of IPN

The potential predictive factors of IPN were analyzed. Eight undetermined patients were excluded from this analysis and would be analyzed later. In the univariate analysis, severe category (56.7 vs. 25.0%, *P*=0.012), positive blood mNGS (86.7 vs. 15.0%, *P*<0.001), positive procalcitonin (56.7 vs. 27.5%, *P*=0.026), lower fibrinogen level (median 4.6 vs. 6.3 g/l, *P*=0.001), and elevated blood urea nitrogen (median 7.4 vs. 4.9 mmol/l, *P*=0.003) were associated with IPN. In the multivariate analysis (Supplementary Table 1, Supplemental Digital Content 2, http://links.lww.com/JS9/B881), positive blood mNGS (OR=60.2; 95% CI: 8.8–413.6; *P*<0.001) and lower fibrinogen level (OR=2.0; 95% CI: 1.1–3.3; *P*<0.05) were identified as independent predictors associated with IPN. Positive procalcitonin was not associated with IPN in febrile ANP.

### Predictors of mortality

The potential predictive factors of mortality in patients with febrile ANP were also analyzed. In the univariate analysis, severe category (100 vs. 30.6%, *P*<0.001), positive blood mNGS (81.3 vs. 40.3%, *P*=0.005), positive procalcitonin (87.5 vs. 33.9%, *P*<0.001), and positive blood culture (50.0 vs. 12.9%, *P*=0.003) were associated with increased mortality. In the multivariate analysis (Supplementary Table 2, Supplemental Digital Content 2, http://links.lww.com/JS9/B881), positive procalcitonin (OR=11.7; 95% CI: 2.1–69.4; *P*=0.006) and positive blood culture (OR=4.6; 95% CI: 1.0–20.2; *P*=0.045) were identified as independent predictors associated with increased mortality.

### Undetermined patients

Eight patients (10.3%) who died of irreversible POF before any surgical intervention could be attempted were classified undetermined. Peripancreatic specimens were not available to pathogenically confirm the diagnosis of IPN or SPN. The median survival time in this subgroup was 18 days (18.0–20.5 days). Among them, six cases (75.0%) were positive of blood mNGS, and three cases were positive of both blood mNGS and blood culture. The median procalcitonin level was as high as 18.5 ng/ml (6.3– 47.4 ng/ml).

### Overuse and misuse of real-world antimicrobial therapy

**Figure 3:**
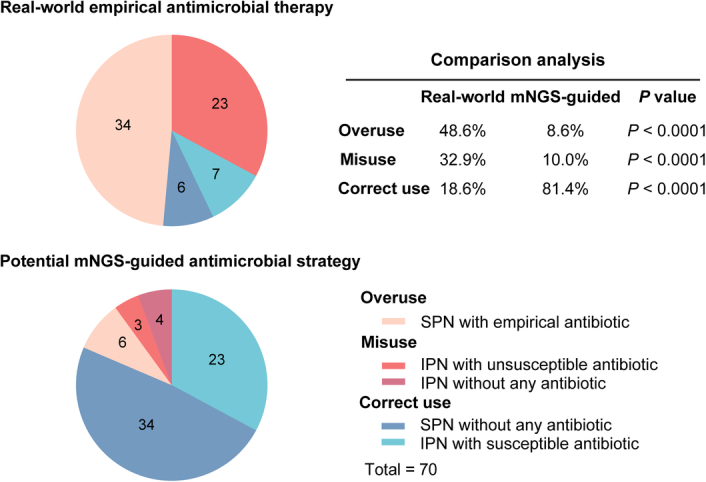
Comparison of real-world empirical antimicrobial therapy and potential mNGS-guided antimicrobial strategy for febrile ANP. ANP, acute necrotizing pancreatitis; IPN, infected pancreatic necrosis; mNGS, metagenomic next-generation sequencing; SPN, sterile pancreatic necrosis.

Empirical antimicrobial therapy was administered to 72 patients (72/78, 92.3%), including 30 patients with IPN (30/30, 100%), 34 patients with SPN (34/40, 85.0%), and eight undetermined patients (8/8, 100%). Third-generation cephalosporins were the most prescribed antibiotics (*n*=26, 36.1%), followed by piperacillin/tazobactam sodium (*n*=20, 27.8%), imipenem/cilastatin (*n*=13, 18.1%), meropenem (*n*=8, 11.1%), ertapenem (*n*=4, 5.6%), and vancomycin (*n*=1, 1.4%). Among the 30 patients with IPN who had empirical antimicrobial therapy, antibiotics provided coverage in seven patients (correct use) and no coverage in 23 patients (misuse). Thirty-four patients with SPN who had empirical antibiotics were classified as overuse of antibiotics and the other six patients with SPN who were not prescribed empirical antibiotics were also classified as correct use (Fig. 3). Overall, the rate of correct use of antibiotics in the cohort was only 18.6% (13/70).

### The potential mNGS-guided antimicrobial strategy in febrile ANP

**Figure 4:**
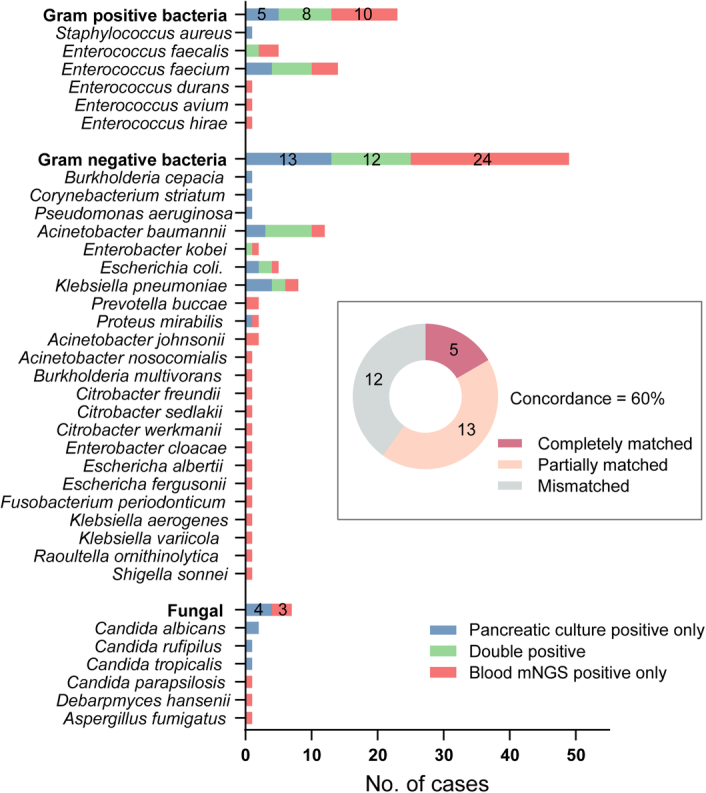
The detailed consistency of pathogens between blood mNGS and peripancreatic culture. mNGS, metagenomic next-generation sequencing; No., number.

This was a real-world observational study and the results of mNGS were blinded to the multidisciplinary teams. Thus, the rates of correct use, overuse, and misuse of antimicrobial therapy were assumed according to the results of mNGS (Fig. 3). Thirty-four SPN patients with negative blood mNGS would not receive antibiotics (correct use) and the remaining six SPN patients with positive blood mNGS would be prescribed antibiotics (overuse). Among the 30 patients with IPN, antibiotics which could provide coverage according to the recommendations from antimicrobial drug usage card by BGI China would be prescribed in 23 patients (correct use). Three patients would be prescribed antibiotics without any coverage to the microorganisms (misuse) and four patients would not be prescribed any antibiotics due to negative mNGS results (misuse). Overall, the rate of correct use of antibiotics would be improved to 81.4% (57/70). The detailed consistency of pathogens between blood mNGS and peripancreatic culture was illustrated in Figure 4.

## Discussion

To our knowledge, this is the first prospective multicenter study to systemically evaluate the effectiveness of blood mNGS in guiding antimicrobial therapy for ANP. This study demonstrated the superiority of blood mNGS over the widely used biochemical ‘gold standard’ procalcitonin as a distinct tool for diagnosis of IPN and initiation of antibiotics in febrile ANP patients. These findings strongly support an important role of mNGS use in helping antibiotic stewardship in AP and may lead to modifications of current practice and even recommendations in guidelines.

In the present study, patient recruitment was restricted to cases with ANP when temperature was more than 38.5°C. In such situation, nearly all the physicians would collect blood samples for culture and inflammatory biomarkers, including procalcitonin, to help establishing or excluding pancreatic infection. Procalcitonin looked like a good solution and seemed to have sound evidence from PROCAP trial^13^. The single-center, patient-blinded, randomized controlled trial demonstrated that procalcitonin-guided care could effectively reduce antibiotic usage in patients with AP without increasing the risk of infection^13^. However, there was no statistically significant risk difference of antibiotic use in patients with severe or febrile ANP who had the higher risk of infective complications^14,15,23^. Rau *et al*.^12^ demonstrated in a prospective international multicenter study that procalcitonin did not allow the prediction of pancreatic infections in general, though procalcitonin proved to be an excellent variable to assess overall prognosis of AP, which was also observed in the present study. Thus, in critically ill patients with ANP, procalcitonin measurements needed to be carefully interpreted. In addition, procalcitonin was a nonspecific marker of sepsis and did not provide any information about the underlying microbes of infection. Whether antibiotics ought to be initiated and what kinds of antibiotics should be used were still unanswered in febrile ANP. Previous studies have observed an excessive and inappropriate utilization of antibiotics in patients with ANP^4,24^. Similar results were also observed in the present study. As high as 92.3% of patients with febrile ANP were administered empirical antibiotics, but only 18.6% were correct use. Early diagnosis of IPN remained to be a significant challenge^25^.

mNGS was an innovative technique that has been utilized for diagnosing various infectious diseases, particularly in cases of rare infection, complicated infection, and critical infection^16,26^. It could provide sensitive and accurate pathogen results in a rapid manner and has been proven to reduce morbidity in acutely ill infants and lower the cost of hospitalization^27^. Our previous study conducted at a single-center with a relatively small sample size showed that blood mNGS had a good potential to be an effective method for early diagnosis of pancreatic infections^18^. However, the clinical effectiveness of mNGS-guided antimicrobial therapy in ANP has not yet been established.

One of the main aims of the present study was to compare blood mNGS and the conventional workup including procalcitonin and blood culture in terms of effectiveness of diagnosing IPN in patients with febrile ANP. The results demonstrated that blood mNGS had a better diagnostic efficacy compared with its counterparts. Compared with procalcitonin and blood culture, not only did mNGS show a significantly higher sensitivity rate, but also outperformed in terms of NPV. The overall accuracy in diagnosing IPN and SPN was also promoted from 65.7% (procalcitonin) and 61.4% (blood culture) to 85.7% (blood mNGS). Since infection sources other than the abdomen such as pulmonary, urinary tract, or catheter infections were frequently observed in critically ill patients, a positive mNGS result needed to be carefully interpreted. However, 81.3% of PPV in the current series, in general, meant in the absence of an abdominal septic focus, other sites of infections had by far a lesser influence on blood mNGS.

The most important strength of the present study was that the usefulness of blood mNGS in guiding antibiotic usage for patients with febrile ANP was assumed and evaluated. With high NPV of 89.5%, blood mNGS showed excellent performance in diagnosing SPN and might help avoid unnecessary antibiotics usage. Moreover, with good concordance of pathogen identification, blood mNGS could greatly facilitate antibiotics choices and decrease antibiotics misuse. Even with great advantages mentioned above, it was noteworthy that due to false negative results of mNGS, four cases of IPN in the study would miss the time window of initiating antibiotics.

According to our previous study^18^, despite the higher cost, mNGS only accounted for 1.8% of the average treatment cost of IPN patients and 3.6% of the average treatment cost of SPN patients. Moreover, the implication of the technique would lead to early diagnosis of infection and timely initiation of infection control strategies including antibiotics and surgical drainage and/or debridement. Consequently, overall hospitalization costs might be reduced accordingly. Furthermore, as the mNGS technique continues to develop and popularize, cost reduction will be anticipated.

There are several limitations to consider. First, the observational nature of this study has inherent limitation in the ability to ascertain the role of mNGS in guiding antibiotic usage in ANP. Randomized controlled clinical trial is warranted in the future. Secondly, there may be IPN patients who have recovered just on antibiotics without any intervention and would have been branded as SPN. Ideal criteria for SPN or IPN population are to obtain culture through FNA of the necrosis. Unfortunately, in the present study, unless patients undergo drainage or surgery, it is not clear who have real pancreatic infection. Given the fact that a fraction (~20–30%) of patients respond to antibiotics alone^28,29^, IPN patients who recovered with antibiotics may have been misclassified as SPN. In addition, we believe that in the undetermined group, most of the patients have a high likelihood of being infected, as indicated by the fact that 75% of patients in this subgroup have positive blood mNGS results and 37.5% are positive of both blood mNGS and blood culture. Thirdly, the risk of false negative of mNGS cannot be ignored. Four cases of IPN in this series presenting with negative blood mNGS may miss the time window of lifesaving antimicrobial therapy if following mNGS-guided antimicrobial therapy alone. Thus, it is important to emphasize that mNGS is no substitute for careful history and clinical examination of the individual patient. Evidence generated from this study supports that mNGS measurement should be added into the armamentarium of guiding antimicrobial therapy for ANP.

In conclusion, it is a prospective multicenter real-world study that highlights applying blood mNGS to early pathogenic diagnosis of IPN. It also emphasizes the significant role of blood mNGS in guiding antibiotic usage for patients with febrile ANP. Further randomized controlled trials are warranted to gain a more comprehensive and definitive conclusion.

## Ethical approval

The study protocol was reviewed and approved by the ethics committee of Xiangya Hospital, Central South University (reference: 202304286) and registered by the ethics committees of all the participating hospitals.

## Consent

Written informed consent was obtained from all patients or their representative for the study participation.

## Sources of funding

This study was supported financially by the Natural Science Foundation of Hunan Province (2023JJ30885) and Postdoctoral Fellowship Program of CPSF (GZB20230872).

## Author contribution

C.L.: conceptualization, data curation, project administration, validation, writing – original draft, and writing – review and editing; J.L.: conceptualization, methodology, formal analysis, software, writing – review and editing; B.L.: data curation, visualization, writing – review and editing; X.H.: data curation, funding acquisition, resources, writing – review and editing; T.L.: data curation, resources, writing – review and editing; J.Y., Y.Y., X.P., S.G., H.T., T.Y., J.L., M.Y., B.F., and Z.Z.: data curation, writing – review and editing; C.N., Z.S., S.Z., and L.C.: supervision, writing – review and editing; D.S.: conceptualization, supervision, resources, writing – review and editing, project administration; G.H.: conceptualization, supervision, resources, funding acquisition, writing – review and editing, project administration. All authors approved the final version of the manuscript.

## Conflicts of interest disclosure

The authors declare that they have no known competing financial interests or personal relationships that could have appeared to influence the work reported in this paper.

## Research registration unique identifying number (UIN)

1. Name of the registry: Chinese Clinical Trial Registry (ChiCTR).
2. Unique identifying number or registration ID: ChiCTR2300071292.
3. Hyperlink to your specific registration (must be publicly accessible and will be checked): https://www.chictr.org.cn/.

## Guarantor

Gengwen Huang and Dingcheng Shen.

## Data availability statement

Data, analytical methods, and study materials are available to other researchers under specific request. The data collected for the study, including anonymous individual participant data and the data dictionary defining each field in the set, will be made available to others for scientific purposes. The data and related documents including analytical methods, and study materials will be available after the publication date on specific request to huanggengwen@csu.edu.cn, with a signed data access agreement and restriction of publication without the authors’ consent.

## Provenance and peer review

Not commissioned, externally peer-reviewed.

## Supplementary Material

## References

[1] Iannuzzi JP King JA Leong JH. Global incidence of acute pancreatitis is increasing over time: a systematic review and meta-analysis. Gastroenterology 2022;162:122–134. 34571026 10.1053/j.gastro.2021.09.043

[2] Huang H Peng J Ning C. Escherichia coli infection indicates favorable outcomes in patients with infected pancreatic necrosis. Front Cell Infect Microbiol 2023;13:1107326. 37051298 10.3389/fcimb.2023.1107326 PMC10083358

[3] Párniczky A Lantos T Tóth EM. Antibiotic therapy in acute pancreatitis: From global overuse to evidence based recommendations. Pancreatology 2019;19:488–499. 31068256 10.1016/j.pan.2019.04.003

[4] Severino A Varca S Airola C. Antibiotic utilization in acute pancreatitis: a narrative review. Antibiotics (Basel) 2023;12:1120. 37508216 10.3390/antibiotics12071120 PMC10376815

[5] Ning C Zhu S Zhou S. Multiple organ failure might be an indication for prophylactic antifungal therapy in acute pancreatitis. Infection 2021;49:769–774. 33988828 10.1007/s15010-021-01625-6

[6] Ning C Huang G Shen D. Adverse clinical outcomes associated with multidrug-resistant organisms in patients with infected pancreatic necrosis. Pancreatology 2019;19:935–940. 31558390 10.1016/j.pan.2019.09.008

[7] Baron TH DiMaio CJ Wang AY. American Gastroenterological Association clinical practice update: management of pancreatic necrosis. Gastroenterology 2020;158:67–75.e1. 31479658 10.1053/j.gastro.2019.07.064

[8] Uhl W Warshaw A Imrie C. IAP Guidelines for the surgical management of acute pancreatitis. Pancreatology 2002;2:565–573. 12435871 10.1159/000071269

[9] Li J Lin C Ning C. Early-onset emphysematous pancreatitis indicates poor outcomes in patients with infected pancreatic necrosis. Dig Liver Dis 2022;54:1527–1532. 35450815 10.1016/j.dld.2022.04.001

[10] Tujula B Hämäläinen S Kokki H. Review of clinical practice guidelines on the use of procalcitonin in infections. Infect Dis (Lond) 2020;52:227–234. 31858869 10.1080/23744235.2019.1704860

[11] Rawson TM Moore LSP. Understanding how diagnostics influence antimicrobial decision-making is key to successful clinical trial design. Clin Microbiol Infect 2023;29:666–669. 36918143 10.1016/j.cmi.2023.03.010 PMC10008184

[12] Rau BM Kemppainen EA Gumbs AA. Early assessment of pancreatic infections and overall prognosis in severe acute pancreatitis by procalcitonin (PCT): a prospective international multicenter study. Ann Surg 2007;245:745–754. 17457167 10.1097/01.sla.0000252443.22360.46 PMC1877072

[13] Siriwardena AK Jegatheeswaran S Mason JM PROCAP investigators. A procalcitonin-based algorithm to guide antibiotic use in patients with acute pancreatitis (PROCAP): a single-centre, patient-blinded, randomised controlled trial. Lancet Gastroenterol Hepatol 2022;7:913–921. 35863358 10.1016/S2468-1253(22)00212-6

[14] Mir BA Majeed T Chauhan A. Procalcitonin-guided use of antibiotics in acute pancreatitis. Lancet Gastroenterol Hepatol 2022;7:1073. 10.1016/S2468-1253(22)00271-0 36370735

[15] Samanta J Dhar J. Procalcitonin-guided use of antibiotics in acute pancreatitis. Lancet Gastroenterol Hepatol 2022;7:1073–1074. 10.1016/S2468-1253(22)00272-2 36370734

[16] Han D Li Z Li R. mNGS in clinical microbiology laboratories: on the road to maturity. Crit Rev Microbiol 2019;45:668–685. 31691607 10.1080/1040841X.2019.1681933

[17] Miao Q Ma Y Wang Q. Microbiological diagnostic performance of metagenomic next-generation sequencing when applied to clinical practice. Clin Infect Dis 2018;67:S231–S240. 30423048 10.1093/cid/ciy693

[18] Lin C Bonsu AAFK Li J. Application of metagenomic next-generation sequencing for suspected infected pancreatic necrosis. Pancreatology 2022;22:864–870. 35864066 10.1016/j.pan.2022.07.006

[19] Hong D Wang P Chen Y. Detection of potential pathogen in pancreatic fluid aspiration with metagenomic next-generation sequencing in patients with suspected infected pancreatic necrosis. Dig Liver Dis 2023;55:243–248. 35948458 10.1016/j.dld.2022.07.014

[20] Mathew G Agha R for the STROCSS Group. STROCSS 2021: strengthening the reporting of cohort, cross-sectional and case-control studies in surgery. Int J Surg 2021;96:106165. 34774726 10.1016/j.ijsu.2021.106165

[21] Banks PA Bollen TL Dervenis C. Classification of acute pancreatitis--2012: revision of the Atlanta classification and definitions by international consensus. Gut 2013;62:102–111. 23100216 10.1136/gutjnl-2012-302779

[22] The European Committee on Antimicrobial Susceptibility Testing. Breakpoint tables for interpretation of MICs and zone diameters. Version 13.1, 2023. http://www.eucast.org

[23] Shen D Wei Q Huang H. Synchronous organ failure and infected pancreatic necrosis define genuine critical acute pancreatitis. Dig Liver Dis 2021;53:1590–1595. 34503931 10.1016/j.dld.2021.08.016

[24] Timmerhuis HC van den Berg FF Noorda PC. Overuse and misuse of antibiotics and the clinical consequence in necrotizing pancreatitis: an observational multicenter study. Ann Surg 2023;278:e812–e819. 36728517 10.1097/SLA.0000000000005790

[25] Ning C Ouyang H Shen D. Prediction of survival in patients with infected pancreatic necrosis: a prospective cohort study. Int J Surg 2023. published online Oct 18. Online ahead of print. doi:10.1097/JS9.0000000000000844 PMC10871654 37851523

[26] Govender KN Street TL Sanderson ND. Metagenomic sequencing as a pathogen-agnostic clinical diagnostic tool for infectious diseases: a systematic review and meta-analysis of diagnostic test accuracy studies. J Clin Microbiol 2021;59:e02916–e02920. 33910965 10.1128/JCM.02916-20 PMC8373000

[27] Farnaes L Hildreth A Sweeney NM. Rapid whole-genome sequencing decreases infant morbidity and cost of hospitalization. NPJ Genom Med 2018;3:10. 29644095 10.1038/s41525-018-0049-4 PMC5884823

[28] Mouli VP Sreenivas V Garg PK. Efficacy of conservative treatment, without necrosectomy, for infected pancreatic necrosis: a systematic review and meta-analysis. Gastroenterology 2013;144:333–340.e2. 23063972 10.1053/j.gastro.2012.10.004

[29] Garg PK Sharma M Madan K. Primary conservative treatment results in mortality comparable to surgery in patients with infected pancreatic necrosis. Clin Gastroenterol Hepatol 2010;8:1089–1094.e2. 20417724 10.1016/j.cgh.2010.04.011

