# Supplementary material for: Metagenomic next-generation sequencing, instead of procalcitonin, could guide antibiotic usage in patients with febrile acute necrotizing pancreatitis: a multicenter, prospective cohort study
Source: Int J Surg. 2024 Feb 9;110(5):2721–9. doi: 10.1097/JS9.0000000000001162 (PMC11093443; doi:10.1097/JS9.0000000000001162)
Supplement: Supplementary file 2 [file js9-110-2721-s002.docx]

**Supplementary Materials**

| **Contents** | **Numbered pages** |
| --- | --- |
| Supplementary Table 1 | 2 |
| Supplementary Table 2 | 3 |

**Supplementary Table 1.** Predictors of IPN in febrile ANP patients

| **Variables** | **IPN**  (n=30) | **SPN**  (n=40) | **Univariable analysis** | **Multivariable**  **analysis** | |
| --- | --- | --- | --- | --- | --- |
|  |  |  | *p* value | OR (95%CI) | *p* value |
| Severe category | 17 (56.7) | 10 (25.0) | 0.012 | 1.0 (0.2-6.1) | 0.969 |
| Positive blood mNGS | 26 (86.7) | 6 (15.0) | <0.001 | 60.2 (8.8-413.6) | <0.001 |
| Positive procalcitonin | 17 (56.7) | 11 (27.5) | 0.026 | 2.6 (0.4-17.3) | 0.310 |
| Positive blood culture | 8 (26.7) | 5 (12.5) | 0.213 | 0.9 (0.1-6.9) | 0.886 |
| C-reactive protein, mg/L | 154.0 (126.0-202.0) | 129.0 (99.0-204.0) | 0.614 | 1.1 (0.2-5.8) | 0.672 |
| Fibrinogen, g/L | 4.6 (3.8-5.2) | 6.3 (4.8-7.3) | 0.001 | 2.0 (1.1-3.3) | 0.018 |
| Blood urea nitrogen, mmol/L | 7.4 (3.9-21.8) | 4.9 (3.2-7.1) | 0.003 | 1.1 (0.9-1.3) | 0.204 |
| Data are presented as n (%) or median (IQR). IPN=infected pancreatic necrosis. ANP=acute necrotizing pancreatitis. SPN=sterile pancreatic necrosis. mNGS=metagenomic next-generation sequencing. OR=odds ratio. CI=confidence interval. | | | | | |

**Supplementary Table 2.** Predictors of mortality in febrile ANP patients

| **Variables** | **Non-survival** | **Survival** | **Univariable analysis** | **Multivariable**  **analysis** | |
| --- | --- | --- | --- | --- | --- |
|  | (n=16) | (n=62) | *p* value | OR (95%CI) | *p* value |
| Severe category | 16 (100) | 19 (30.6) | <0.001 | 1.7 (0.4-10.0) | 0.480 |
| Positive blood mNGS | 13 (81.3) | 25 (40.3) | 0.005 | 4.7 (0.8-28.1) | 0.093 |
| Positive procalcitonin | 14 (87.5) | 21 (33.9) | <0.001 | 11.7 (2.1-69.4) | 0.006 |
| Positive blood culture | 8 (50.0) | 8 (12.9) | 0.003 | 4.6 (1.0-20.2) | 0.045 |
| Data are presented as n (%). ANP=acute necrotizing pancreatitis. mNGS=metagenomic next-generation sequencing. OR=odds ratio. CI=confidence interval. | | | | | |
